# Performance of the EWGSOP2 Cut-Points of Low Grip Strength for Identifying Sarcopenia and Frailty Phenotype: A Cross-Sectional Study in Older Inpatients

**DOI:** 10.3390/ijerph18073498

**Published:** 2021-03-28

**Authors:** Anna K. Stuck, Nina C. Mäder, Dominic Bertschi, Andreas Limacher, Reto W. Kressig

**Affiliations:** 1 Department of Geriatrics, Inselspital, Bern University Hospital, University of Bern, 3010 Bern, Switzerland; dominic.bertschi@bluewin.ch; 2 Medical Faculty, University of Bern, 3012 Bern, Switzerland; nina_maeder@bluewin.ch; 3 CTU Bern, University of Bern, 3012 Bern, Switzerland; andreas.limacher@ctu.unibe.ch; 4 University Department of Geriatric Medicine FELIX PLATTER, University of Basel, 4055 Basel, Switzerland; RetoW.Kressig@felixplatter.ch

**Keywords:** post-acute care, rehabilitation, geriatric, muscle strength, gait speed, fried phenotype, clinical frailty scale, cut-off value

## Abstract

Background: The European Working Group on Sarcopenia has recently proposed revised cut-off values for the definition of low grip strength (EWGSOP2). We therefore compared performance of the EWGSOP2 cut-off definition of low grip strength with other internationally used cut-off points in a sample of older patients. Methods: We analyzed geriatric assessment data in a cross-sectional sample of 98 older patients admitted to a post-acute care hospital. First, we compared prevalence of sarcopenia and frailty phenotype in our sample using low grip strength cut-points from the EWGSOP2 and seven other internationally used consensus statements. Second, we calculated correlations between low grip strength and two independent surrogate outcomes (i.e., gait speed, and the clinical frailty scale) for the EWGSOP2 and the other seven cut-point definitions. Results: Prevalence of sarcopenia based on the EWGSOP2 grip strength cut-off values was significantly lower (10.2%) than five of the seven other cut-point definitions (e.g., 19.4% based on Sarcopenia Definitions and Outcomes Consortium (SDOC) criteria). Similarly, frailty phenotype prevalence was significantly lower based on EWGSOP2 cut-points (57.1%) as compared to SDOC (70.4%). The correlation coefficient of gait speed with low grip strength based on EWGSOP2 cut-points was lower (0.145) as compared to other criteria (e.g., SDOC 0.240). Conclusions: Sarcopenia and frailty phenotype were identified considerably less using the EWGSOP2 cut-points for low grip strength, potentially underestimating prevalence of sarcopenia and frailty phenotype in post-acute hospital patients.

## 1. Introduction

Sarcopenia and physical frailty are clinical syndromes occurring in older patients associated with adverse outcomes such as mobility impairment, fracture, functional impairment, and mortality [1,2]. Prior evidence shows that both sarcopenia and frailty are common in older inpatients (42% and 33%) and often even overlap due to similar etiological pathways [3]. It is therefore important to identify sarcopenia and frailty in older patients for implementing targeted interventions to prevent adverse clinical outcomes.

In the diagnostic work-up of sarcopenia, frailty grip strength is a core determinant as a proxy measurement for overall muscle strength. In the latest guidelines of the European Working Group on Sarcopenia in Older People (EWGSOP2), low grip strength is the primary parameter together with low muscle mass to diagnose sarcopenia [4]. Similarly, low grip strength is one of the five criteria, along with shrinking, exhaustion, slowness, and low activity, for defining the Fried frailty phenotype [5].

The EWGSOP2 consensus statement proposes new cut-points for defining low grip strength [4]. These cut-points are higher than those defined by the EWGSOP1 published in 2010 [6] and higher than other internationally used cut-points, including the most recent cut-points recommended by the Sarcopenia Definitions and Outcomes Consortium (SDOC) [7] in 2020 after the EWGSOP2 was published. Moreover, the Asia Working Group on Sarcopenia (AWGS) [8,9], the Foundation for the National Institutes of Health Biomarkers Consortium Sarcopenia Project (FNIH) [10,11], and the Fried definition [5] proposed different cut-off points of low grip strength. To provide an overview, we summarized these seven internationally used definitions of low grip strength and their corresponding derivation studies in Table 1.

The wide range of different published cut-points is confusing, given the importance of grip strength in the diagnosis of sarcopenia and identification of physical frailty. Therefore, using the EWGSOP2 cut-points of low grip strength instead of using other cut-points could have an impact on the prevalence of patients identified with sarcopenia and frailty. However, we were unable to find any studies that have systematically compared detection rates for sarcopenia and frailty between published grip strength cut-points. It is unclear, how many patients with sarcopenia and frailty would be overlooked depending on what cut-point of low grip strength is used.

The purpose of this study was to determine how the EWGSOP2 cut-points for low grip strength compared to other internationally used cut-points perform for identifying sarcopenia and frailty phenotype in a sample of older patients in a post-acute care hospital.

## 2. Materials and Methods

We conducted a retrospective analysis of cross-sectional anonymized assessment data of patients admitted to a Geriatric Rehabilitation Hospital in Bern, Switzerland, between November and December 2019. Standard geriatric assessments were conducted for all patients upon admission. The assessments were performed by clinically trained assessors (two medical students in their last semester before graduation) using standardized assessment forms. We excluded 9 patients who had a contraindication for muscle mass measurement by bioelectrical impedance analyses (implantable cardioverter defibrillator or pacemaker), leaving a total sample of 98 patients (65.7% female, mean age 84 years (sd 5.8)) included in the analysis.

Our study project was approved by the Ethics Committee in Bern, Switzerland (Req-2020-00125). In accordance to regulations on research projects of health-related data of human beings in Switzerland we analyzed anonymized data. While researchers do work with health-related data of human beings, the data can no longer be assigned to the specific individual.

### 2.1. Sarcopenia and Frailty Assessment

On admission, patients were assessed for sarcopenia and frailty. The assessment of sarcopenia was based on the EWGSOP2 consensus guidelines [4] and included measurement of appendicular skeletal muscle mass, grip strength, and gait speed. Appendicular skeletal muscle mass was measured upon admission using bioelectrical impedance analysis (BIACORPUS RX 4004M, MEDI CAL HealthCare GmbH). Low muscle mass was defined according to the EWGSOP2 gender-specific cut-off definitions of the appendicular skeletal muscle mass index (women < 5.5 kg/m^2^, men < 7 kg/m^2^) [4].

Grip strength was assessed in an upright sitting position using the Martin vigorimeter. The test was performed 3 times using the dominant hand, with a 30 s interval between each measurement. Patients were instructed to firmly squeeze the balloon for three seconds. The maximum score of the 3 measures was used for analysis. Scores were converted from kPa to kg using the conversion table by Neumann et al. [15].

Gait speed was measured using a standardized protocol of a 4m walk test [16,17]. Patients were instructed to walk 4 m (standing start, manual timing) at their usual speed. The test was repeated twice with a rest time of 30 s in between and the best of the two consecutive measurements was used for analysis. Patients were allowed to use an assistive device (e.g., walking cane, frame), however, if the patient had limited mobility capacity (<10 m walking distance without aid) or needed personal assistance, gait speed was recorded as 0 m/s.

Frailty was assessed using Fried’s frailty phenotype [5]. The frailty phenotype classifies older people as frail based on five characteristics (i.e., shrinking, low activity, fatigue, slowness, weakness). Scoring definitions for each characteristic are shown in the Supplemental Information. A score ≥ 3 out of 5 is considered positive for the frailty phenotype. The clinical frailty scale (CFS), another measure of frailty not including measurement of grip strength, was also completed for each patient according to the definition by Rockwood et al. [18]. The CFS is an ordinal scale ranging from 1 (least frail) to 9 (most frail).

### 2.2. Low Grip Strength Cut-Points

In addition to the EWGSOP2, we identified low grip strength cut-points from seven other consensus definitions of sarcopenia and frailty phenotype. These included (listed in alphabetic order):

(1) Asia Working Group for Sarcopenia (AWGS1) [8].
(2) Asia Working Group for Sarcopenia (AWGS2) [9].
(3) European Working Group on Sarcopenia in Older People 1 (EWGSOP1) [6].
(4) The Foundation for the National Institutes of Health Biomarkers Consortium Sarcopenia Project 1 (FNIH 1) [10].
(5) The Foundation for the National Institutes of Health Biomarkers Consortium Sarcopenia Project 2 (FNIH 2) [11].
(6) Fried frailty phenotype (Fried) [5].
(7) Sarcopenia Definitions and Outcomes Consortium, (SDOC) [7].

### 2.3. Statistical Analyses

Study population characteristics from admission data are presented by absolute and relative frequencies or by mean with standard deviation (sd) for categorical and continuous variables, respectively. Power analysis was based on prior evidence expecting prevalence of sarcopenia of 20% in older patients in a post-acute care setting [19]. At a two-sided confidence level of 0.05, the sample size of 98 patients yields a precision of +/− 8% [20]. McNemar testing was used to compare prevalence of sarcopenia and frailty phenotype using the EWGOSP2 cut-off definitions of low grip strength versus the other cut-off definitions of low grip strength. Adjusted *p*-values (*q*-value) of McNemar tests using the Hochberg method were calculated to account for multiple pairwise comparisons. The decision to not perform statistical comparisons among subgroups of patients to avoid type I and II error inflation was made a priori. To assess convergent validity, we investigated correlations between different low grip strength cut-point with independent outcomes of sarcopenia (gait speed) and frailty (clinical frailty scale (CFS)). The point biserial correlation coefficient was calculated to analyze correlation of prevalence of low grip strength based on cut-off definitions (binary variables) with gait speed (continuous variable) and CFS (ordinal scale). All analyzes were computed using Stata Version 16.0 (StataCorp LLC, College Station, TX, USA). An adjusted p-value of <0.05 was considered statistically significant.

## 3. Results

### 3.1. Prevalence of Low Grip Strength, Sarcopenia, and Frailty Phenotype

The overall and gender-specific clinical characteristics of the sample are summarized in Table 2.

The prevalence of low grip strength, sarcopenia, and frailty phenotype calculated using the EWGSOP2 cut-off and for each of the other seven cut-off definitions of low grip strength is compared in Table 3.

Overall, the EWGSOP2 cut-off value yielded the lowest prevalence of low grip strength (27.6%), while the SDOC cut-off definition yielded the highest prevalence (56.1%). All cut-off definitions, except for those used by the AWGS1 and FNIH1, showed statistically significant higher prevalence of low grip strength compared with the EWGSOP2 cut-off definition (*p* < 0.01).

Prevalence of sarcopenia based on the EWGSOP2 cut-points of low grip strength was significantly lower than those based on the SDOC, FNIH2, and EWGSOP1 definitions (Table 3). Similarly, frailty phenotype prevalence was significantly lower using the EWGSOP2 cut-off values compared with SDOC, FNIH2, and EWGSOP1 cut-off definitions.

Gender-specific prevalence of low grip strength, sarcopenia, and frailty phenotype is outlined in Table 4. Prevalence of sarcopenia ranged from 9.4 to 28.1% in men, whereas in women prevalence ranged from 9.1 to 15.2%. This section may be divided by subheadings. It should provide a concise and precise description of the experimental results, their interpretation, as well as the experimental conclusions that can be drawn.

### 3.2. Correlations with Surrogate Outcomes

Table 5 displays correlations between different low grip strength cut-points with independent outcomes of sarcopenia (gait speed) and frailty (CFS). The correlation of gait speed with CFS based on EWGSOP2 was lower (0.167) than correlations with low grip strength based on the other criteria (e.g., SDOC 0.257). The cut-off definition based on EWGSOP2 showed the lowest correlation with gait speed (0.145) compared with the other cut-off definitions.

## 4. Discussion

We found that within a sample of post-acute hospital patients, the prevalence estimates of sarcopenia and frailty phenotype based on the EWGSOP2 cut-off definition of low grip strength were significantly lower than estimates obtained from other recommended cut-off definitions. These results suggest that the EWGSOP2 cut-off definition of low grip strength potentially underestimates prevalence of sarcopenia and frailty phenotype.

To our knowledge, this is the first study comparing cut-off definitions of low grip strength to define prevalence of sarcopenia and frailty phenotype. A previous study [21] investigated the effect of the EWGSOP2 consensus on prevalence of sarcopenia in a sample of older inpatients, but did not compare the impact of the grip strength cut-off definitions on sarcopenia prevalence. After applying the EWGSOP2 and the EWGSOP1 criteria to their study sample, van Ancum et al. [21] found lower prevalence of sarcopenia with the newer consensus recommendations. The authors concluded that the EWGSOP2 cut-off values for low grip strength may fail to identify patients with low physical performance and/or low muscle mass. Reiss et al. [22] found that concordance rates of individual cases of sarcopenia were low between EWGSOP1 and EWGSOP2. Another study did not focus on EWGSOP criteria, but compared other cut-off definitions of low grip strength demonstrating that different cut-off definitions of low grip strength result in different proportions of people identified with low grip strength [23]. However, this study [23] did not investigate the impact of cut-off definitions of low grip strength on prevalence of sarcopenia and frailty phenotype. Finally, Bertschi et al. applied EWGSOP2 consensus in sample older inpatients to obtain prevalence of sarcopenia, but did not investigate prevalence of sarcopenia using other definitions [19].

Our findings that the EWGSOP2 cut-off definition of low grip strength had a lower correlation with gait speed and CFS than other recommended cut-points is another important finding. Gait speed used as an independent surrogate of sarcopenia, is considered a key measure in the frame of sarcopenia and is even used as a criterion for diagnosis of sarcopenia in some consensus definitions [9,11,24]. The relevance of gait speed in the frame of sarcopenia is underlined by the fact that several cut-off levels of grip strength (SDOC, FNIH1, FNIH2) were derived from a model best predicting gait speed. Recently, the Sarcopenia Definition and Outcomes Consortium panel even agreed that both weakness defined by low grip strength and slowness defined by low usual gait speed should be included in the definition of sarcopenia [24]. This conclusion was based on a study showing that both muscle weakness and slowness are associated with incident fall, hip fracture, and mobility limitation [25]. The other surrogate measure used for frailty, the CFS, is recognized as a frailty measure independent of grip strength being used in multiple settings and being associated with clinical adverse outcomes [26].

Therefore, the low correlation coefficients of EWGSOP2 cut-points of low grip strength that we found in our study suggest that EWGSOP2 cut-off definitions of low grip strength may have limited validity for identifying cases of sarcopenia and frailty compared with the other cut-off definitions of low grip strength.

Interpretation of these results is challenging because there is no gold standard for identifying patients with true sarcopenia or true frailty. Consequently, we cannot make a conclusive statement about which cut-off definition is most valid to detect sarcopenia and frailty. Nevertheless, our results showing higher convergent validity of SDOC than EWGSOP2 suggest that the use of the SDOC and other similar cut-off definitions more accurately reflect the true prevalence of sarcopenia and frailty in older adult patients.

The lower prevalence rates associated with the EWGSOP2 are most likely due to different methodologies used by studies to derive cut-off points. There is substantial variation between the derivation studies with regard to study populations, how grip strength was measured (e.g., type of device, number of trials, dominant vs. non-dominant hand) and methods for determination of cut-points. EWGSOP2 used a similar approach for derivation of cut-off that is known for the definition of osteoporosis, setting the cut-off at −2.5 SD below the gender-specific mean. In contrast, the SDOC and FNIH based their cut-off for low grip strength on a model best predicting slow walking speed in an older population. This was also the approach used for the EWGSOP1 cut-off definition. EWGSOP2 proposed the lowest cut-off points of low grip strength for both men and women, resulting in low prevalence of sarcopenia and frailty. Similarly, van Ancum et al. concluded that setting the cut-off of low grip strength at −2.5 SD may account for lower prevalence of sarcopenia based on EWGSOP2 [21]. Nevertheless, it remains unclear what derivation approach is best for defining cut-points that consequently lead to the most accurate and valid definition of sarcopenia and frailty.

Subgroup analyses in our study suggest that men demonstrate higher prevalence rates of both frailty and sarcopenia. In our study population, male patients tended to show higher degrees of multimoribidity and polypharmacy, suggesting a generally greater disease burden compared to female patients. Thus, general health status may account for the higher prevalence of sarcopenia and frailty that we observed among men [27].

### 4.1. Limitations

Our study has several limitations. First, our study focused only on how variability in grip strength cut-points impacted the prevalence of sarcopenia and frailty phenotype in one sample of post-acute hospital patients. Other methodological issues associated with measurement of grip strength measurement (e.g, type of device, instructions, selected hand, number of trials, result used for analysis) could also affect prevalence [28]. Second, we did not investigate the impact of other criteria (e.g., muscle mass, shrinking) used for the overall diagnostic work-up of sarcopenia and frailty phenotype. However, we believe that our findings of investigating the impact of one specific aspect—cut-off choice for low grip strength—may contribute to a basis for future consensus definitions. Third, while it is possible that we omitted other cut-off definitions of low grip strength in our comparisons to the EWGSOP2 definition, it is unlikely that these omissions would substantially change our finding that the EWGSOP2 cut-point yields lower prevalence of low grip strength compared to other definitions.

### 4.2. Implications

Our results have both clinical and research implications. Our results demonstrate that the selection of a cut-off point for defining low grip strength has a clinically relevant impact on identification of sarcopenia and frailty phenotype. The EWGSOP2 recommended cut-off for low grip strength results in lower prevalence of sarcopenia and frailty compared with various other internationally used cut-off definitions of low grip strength. Correlation analyses of our data suggest limited validity of EWGSOP2 cut-off definition of low grip strength for identifying sarcopenia and frailty phenotype. Therefore, clinicians should be aware of the fact that patients with an actual sarcopenia or frailty phenotype are potentially overlooked by applying the EWGSOP2 cut-points of low grip strength.

Further research is needed to clarify which cut-off values of low grip strength are adequate for identifying cases of sarcopenia and frailty phenotype in older patients. Ideally for practical use, cut-off values of low grip strength would be the same for the diagnostic work-up of both sarcopenia and frailty phenotypes. Since concepts of sarcopenia as an ICD relevant diagnosis and frailty phenotype as a clinical syndrome are different, it is possible that different cut-off values are preferable for sarcopenia and frailty phenotype. Therefore, interventional and longitudinal studies are needed to identify and agree on a valid cut-off of low grip strength in the context of sarcopenia and frailty phenotype.

## 5. Conclusions

The selection of a cut-point of low grip strength has a clinically relevant impact on the prevalence of sarcopenia and frailty phenotypes. Applying the EWGSOP2 cut-points potentially results in underestimation of actual sarcopenia and frailty phenotype compared with various other cut-off values of low grip strength.

## Figures and Tables

**Table 1:** Characteristics of derivation studies used for consensus definition of low grip strength.

Name of Consensus (YOP)	Study Population Characteristics			Measurement Method of Grip Strength				Method for Derivation of Cut-Offs Points
	Sample Size and Region	Age	Characteristics of Participants	Device Used	No. of Trials per Hand	Selection of Hand	Value Used for Analysis	
EWGSOP2 (2020)	49,964 Great Britain (12 cohorts) [12]	Median: 58 (Range 4–102)	Community-dwelling Exclusion: none	Mixed: Jamar Harpenden Smedley Nottingham Takei	Mixed 2–3	Mixed Both hands, Domi-nant hand	Max.	≥2.5 SDs below the gender-specific population mean
AWGS1 (2014)	1158 Japan [8]	Mean W: 73.9 Mean M: 74.4 (Range 65 to N.r.)	Community-dwelling Exclusion: none	Takei	1	Both hands	Max.	Lowest quartile (by gender)
AWGS2 (2019)	26,344 Asia (7 cohorts) [13]	Mean: N.r. (Range 65 to N.r.)	Community-dwelling Exclusion: none	Mixed: Smedley Jamar	2	N.r.	Max.	Lowest quintile (by gender)
EWGSOP1 (2010)	1030 Italy [14]	Mean: N.r. (Range 20–102)	Community-dwelling Exclusion: Participants stroke, Parkinson’s disease, peripheral neuropathy, and cognitive impairment	N.r.	N.r.	N.r.	N.r.	Optimal cutoff according to ROC in the identification of participants walking slower than 0.8 m/s
FNIH2 (2014)	20,847 USA/ Europe (9 cohorts) [11]	Mean W: 78.1 Mean M: 74.9 (Range 65 to N.r.)	Community-dwelling Exclusion: none	Mixed: Jamar Smith& Nephew Medley	Mixed 2–3	MixedBoth hands, right hand, left hand	Max.	CART analysis predicting probability of slow walking (<0.8 m/s) (by gender) ^(a)^
FNIH1 (2014)	26,625 USA/ Europe (9 cohorts) [10]	Mean W: 78.6 Mean M: 75.2 (Range 65 to N.r.)	Community-dwelling Exclusion: none	Mixed (Jamar, Smith Medley)	N.r.	Either hand	Max.	CART analysis predicting probability of slow walking (<0.8 m/s) (by gender) ^(a)^
Fried (2001)	5317 USA (2 cohorts) [5]	Mean: N.r. (Range 65-101)	Community-dwelling Exclusion: history of Parkinson’s disease, stroke, or Mini-Mental scores <18, and intake of Sinemet, Aricept, or antidepressant	Jamar	3	Domi-nant	Mean	Lowest 20th percentile (by gender, and BMI)
SDOC (2020)	18,767 USA/ Europe/ Asia (8 cohorts) [7]	Mean W: 77.1 Mean M: 78.1 (Range 65 to N.r.)	Community-dwelling Exclusion: none	Jamar	Mixed 2–3	Either hand	Max.	CART analyses (ROC curve and Youden index) predicting slow walking speed (<1.0 m/s)

Abbreviations: N.r., not reported; YOP, year of publication; AWGS, Asia Working Group on Sarcopenia; EWGSOP, European Working Group on Sarcopenia in Older People; FNIH, The Foundation for the National Institutes of Health Biomarkers Consortium Sarcopenia Project; SDOC, Sarcopenia Definitions and Outcomes Consortium; SD, standard deviation; W, women; M, men; ROC, receiver operating curve. ^(a)^ Detailed specification by the authors: “Because the additional value of including BMI in the definition of weakness was unclear (ie, including BMI did not consistently improve model fit or result in stronger associations between weakness and slowness), we elected to use cutpoints based on grip strength alone as our primary analysis.”

**Table 2:** Study population characteristics (*n* = 98).

	Overall	Women	Men
	(*n* = 98)	(*n* = 66)	(*n* =32)
General characteristics			
Age, mean (sd), range (years)	84 (5.8) 70 to 97	84.4 (5.6) 70 to 97	83.3 (6.3) 71 to 95
Weight, mean (sd) (kg)	70.2 (15.6)	66.6 (15.1)	77.6 (14.1)
Height, mean (sd) (cm)	163.7 (8.9)	160.0 (6.6)	171.4 (8.2)
BMI, mean (sd) (kg/m^2^)	26.2 (5.4)	26.0 (5.7)	26.4 (4.8)
Multimorbidity index ^(a)^, median (IQR)	18 (16–21)	17.5 (15–20)	20 (17–21.5)
Functional independence measure (FIM) ^(b)^ median (IQR)	83.5 (69–94)	85.5 (70–95)	81.5 (69–92.5)
Number of medications, mean (sd)	11.6 (5.4)	11.4 (5.4)	12.0 (5.3)
CFS scale, median (IQR)	5 (5–6)	5 (4–6)	6 (5–7)
Gait speed, mean (sd) (m/s)	0.33 (0.28)	0.34 (0.30)	0.30 (0.23)
Frailty phenotype characteristics			
Shrinking, *n* (%)	85 (86.7%)	59 (89.4%)	26 (81.3%)
Exhaustion, *n* (%)	27 (27.6%)	15 (22.7%)	12 (37.5%)
Low activity, *n* (%)	34 (34.7%)	24 (36.4%)	10 (31.3%)
Slowness, *n* (%)	94 (95.9%)	62 (93.9%)	32 (100.0%)
Sarcopenia characteristics			
Low muscle mass	31 (31.6%)	20 (30.3%)	11 (34.4%)

Abbreviations: sd, standard deviation; BMI, body mass index; CFS, clinical frailty scale; IQR, interquartile range; ^(a)^ Cumulative Illness Rating Scale (CIRS). Higher values indicate higher degree of multimorbidity; ^(b)^ higher values indicate higher degree of functional independence.

**Table 3:** Prevalence of low grip strength, sarcopenia, and frailty phenotype based on EWGSOP2 cut-off definition of low grip strength compared with other cut-off definitions of low grip strength (*n* = 98).

Name of Recommendation	Grip Strength Cut-Off Definition	Low Grip Strength, *n* (%)	*p*-Value ^(b)^	Sarcopenia ^(c)^ *n* (%)	*p*-Value ^(b)^	Frailty Phenotype ^(d)^ *n* (%)	*p*-Value ^(b)^
EWGSOP2	Women <16 kg Men <27 kg	27 (27.6%)	Ref	10 (10.2%)	Ref	56 (57.1%)	Ref
AWGS1	Women < 18 kg Men <26 kg	32 (32.7%)	0.12	10 (10.2%)	1.00	56 (57.1%)	1.00
AWGS2	Women <18 kg Men <28 kg	37 (37.8%)	<0.01	12 (12.2%)	0.47	59 (60.2%)	0.25
EWGSOP1	Women <20 kg Men <30 kg	45 (45.9%)	<0.01	18 (18.4%)	0.02	64 (65.3%)	0.02
FNIH1	Women <16 kg Men <26 kg	26 (26.5%)	0.32	9 (9.2%)	0.63	55 (56.1%)	0.63
FNIH2	Women <19.99 kg Men <31.83 kg	50 (51.0%)	<0.01	19 (19.4%)	0.02	67 (68.4%)	<0.01
Fried	Women ≤17 to ≤21kg ^(a)^ Men ≤29 to ≤32 kg ^(a)^	41 (41.8%)	<0.01	13 (13.3%)	0.33	62 (63.3%)	0.06
SDOC	Women <20 kg Men <35.5 kg	55 (56.1%)	<0.01	19 (19.4%)	0.02	69 (70.4%)	<0.01

Abbreviations: AWGS, Asia Working Group for Sarcopenia; SDOC, Sarcopenia Definitions and Outcomes Consortium; EWGSOP, European Working Group on Sarcopenia in Older People; FNIH, The Foundation for the National Institutes of Health Biomarkers Consortium Sarcopenia Project; Ref, Referent. ^(a)^ Detailed cut-off value according to gender and BMI: Women BMI≤ 23: ≤17 kg; BMI 23.1–26: ≤17.3; BMI 26.1-29: ≤18 kg; BMI >29 kg: ≤21 kg. Men BMI ≤24: ≤29 kg; BMI 24.1–26: ≤30 kg; BMI 26.1–28: ≤30 kg; BMI >28: ≤32 kg. ^(b)^ McNemar test comparing proportions of each grip strength definition versus EWGSOP2 grip strength definition, adjusted for multiple pairwise testing (*q*-value) using the Hochberg method. ^(c)^ Sarcopenia is defined as low grip strength (using the indicated cut-off definition) and low muscle mass (using the gender-specific cut-off value for low muscle mass according to EWGSOP2). ^(d)^ Fried frailty phenotype according to definition in Methods section.

**Table 4:** Prevalence of low grip strength, sarcopenia, and frailty phenotype based on EWGSOP2 cut-off definition of low grip strength compared with other cut-off definitions of low grip strength, by gender (*n* = 98).

Name of Grip Consensus	Grip Strength Cut-Off Definition	Women (*n* = 66) ^(a)^			Men (*n* = 32) ^(a)^		
		Low Grip Strength	Sarcopenia ^(b)^	Frailty Phenotype ^(c)^	Low Grip Strength	Sarcopenia ^(b)^	Frailty Phenotype ^(c)^
EWGSOP2	Women <16 kg Men <27 kg	16 (24.2%)	6 (9.1%)	37 (56.1%)	11 (34.4%)	4 (12.5%)	19 (59.4%)
AWGS1	Women <18 kg Men <26 kg	22 (33.3%)	7 (10.6%)	38 (57.6%)	10 (31.3%)	3 (9.4%)	18 (56.3%)
AWGS2	Women <18 kg Men <28 kg	22 (33.3%)	7 (10.6%)	38 (57.6%)	15 (46.9%)	5 (15.6%)	21 (65.6%)
EWGSOP1	Women <20 kg Men <30 kg	26 (39.4%)	10 (15.2%)	40 (60.6%)	19 (59.4%)	8 (25.0%)	24 (75.0%)
FNIH1	Women <16 kg Men <26 kg	16 (24.2%)	6 (9.1%)	37 (56.1%)	10 (31.3%)	3 (9.4%)	18 (56.3%)
FNIH2	Women <20 kg Men <31.8 kg	26 (39.4%)	10 (15.2%)	40 (60.6%)	24 (75.0%)	9 (28.1%)	27 (84.4%)
Fried	Women ≤17 to ≤21 kg ^(d)^ Men ≤29 to ≤32 kg ^(d)^	22 (33.3%)	6 (9.1%)	38 (57.6%)	19 (59.4%)	7 (21.9%)	24 (75.0%)
SDOC	Women <20 kg Men <35.5 kg	26 (39.4%)	10 (15.2%)	40 (60.6%)	29 (90.6%)	9 (28.1%)	29 (90.6%)

Abbreviations: SDOC, Sarcopenia Definitions and Outcomes Consortium; AWGS; Asia Working Group on Sarcopenia; EWGSOP, European Working Group on Sarcopenia in Older People; FNIH, The Foundation for the National Institutes of Health Biomarkers Consortium Sarcopenia Project. ^(a)^ Statistical testing of subgroups is not performed to avoid type-I and –II error inflation. ^(b)^ Sarcopenia is defined as low grip strength (using the indicated cut-off definition) and low muscle mass (using the gender-specific cut-off value for low muscle mass according to EWGSOP2). ^(c)^ Fried frailty phenotype according to definition in Methods section. ^(d)^ Cut-points of low grip strength dependent on body mass index (Women BMI ≤23: ≤17 kg; BMI 23.1–26: ≤17.3; 26.1–29: ≤18 kg; >29 kg: ≤21 kg; Men BMI ≤24: <=29 kg; BMI 24.1–26: ≤30 kg; BMI 26.1–28: ≤30 kg; BMI >28: ≤32 kg).

**Table 5:** Correlation coefficients (r) of cut-off definitions of low grip strength with clinical frailty scale (CFS) and gait speed (*n* = 98).

Name of Consensus	Correlation with CFS ^(a)^	Correlation with Gait Speed ^(b)^
EWGSOP2	0.167	0.145
AWGS1	0.178	0.259
AWGS2	0.228	0.251
EWGSOP1	0.201	0.213
FNIH1	0.177	0.168
FNIH2	0.164	0.233
Fried	0.268	0.243
SDOC	0.257	0.240

Abbreviations: CFS, clinical frailty scale; AWGS; Asia Working Group on Sarcopenia; EWGSOP, European Working Group on Sarcopenia in Older People; FNIH, The Foundation for the National Institutes of Health Biomarkers Consortium Sarcopenia Project; SDOC, Sarcopenia Definitions and Outcomes Consortium. ^(a)^ Positive correlation coefficients (r) indicate low grip strength correlating with frailty on the CFS (ordinal scale). ^(b)^ Correlation coefficients (r) were reversed to enable better reading: Positive correlation coefficients indicate low grip strength correlating with slower gait speed (continuous scale).

## Data Availability

The data presented in this study are available in the article.

## Supplementary Materials

The following are available online at https://www.mdpi.com/article/10.3390/ijerph18073498/s1, Table S1: Definition of Fried frailty phenotype.
