# Supplementary material for: Performance of the EWGSOP2 Cut-Points of Low Grip Strength for Identifying Sarcopenia and Frailty Phenotype: A Cross-Sectional Study in Older Inpatients
Source: Int J Environ Res Public Health. 2021 Mar 28;18(7):3498. doi: 10.3390/ijerph18073498 (PMC8037004; doi:10.3390/ijerph18073498)
Supplement: Supplementary file 1 [file ijerph-18-03498-s001.pdf]

Supplementary information

For the article entitled:

Performance of the EWGSOP2 cut-points of low grip strength for identifying sarcopenia and frailty phenotype: A cross-sectional study in older inpatients

Authors:

Anna K. Stuck, Nina C. Mäder, Dominic Bertschi, Andreas Limacher, Reto W. Kressig

January 30, 2021

Table S1. Definition of Fried frailty phenotype

1) Shrinking: Unintentional weight loss >5% during last 6 months, or loss of appetite, or loose clothing. If the patient had a cognitive impairment (MMS ≤16 points), proxy information was collected. If there was no proxy available, a frailty point was attributed if the nutritional assessment (daily total nutritional intake evaluated upon admission) was graded insufficient.

2) Low activity: The patient has limited mobility (<10m walking distance) and needs personal assistance for walking (walking aid permitted), OR the patient reports to engage activities that require a low or moderate level of energy such as gardening, cleaning a car, or doing a walk for "less than once a week" before admission to the hospital. If the patient had a cognitive impairment (MMS ≤24 points), proxy information was collected.

3) Fatigue: Self-reported 5-item Geriatric depression scale (5-GDS) ≥2 points. If the patient was cognitively impaired (MMS ≤16 points) the complete list of medication was screened for ≥1 antidepressive substance.

4) Slowness: <0.8m/sec measured on a standardized 4m gait speed test. Patients were asked to walk 4m (standing start, manual timing) at their usual speed. The 4-m walk test was repeated twice and patients were allowed to use an assistive device (the best of two consecutive measurements was used for analysis). If the patient had limited mobility capacity (<10m walking distance) and needed personal assistance gait speed of 0m/sec was recorded.

5) Weakness: Grip strength using a Martin vigorimeter at the dominant hand at the time of assessment. Three trials were performed and the maximum value achieved was used for analysis. Weakness was defined using the cut-off definitions of low grip strength according to the Fried frailty phenotype (Women BMI ≤23: ≤17kg; BMI 23.1-26: ≤17.3; 26.1-29: ≤18kg; >29kg: ≤21kg; Men BMI ≤24: ≤29kg; BMI 24.1-26: ≤30kg; BMI 26.1-28: ≤30kg; BMI >28: ≤32kg).
